# Thirty-day rehospitalizations among elderly patients with acute myocardial infarction: Impact of postdischarge ambulatory care

**DOI:** 10.1097/MD.0000000000011085

**Published:** 2018-06-15

**Authors:** Claire Zabawa, Jonathan Cottenet, Marianne Zeller, Grégoire Mercier, Victor G. Rodwin, Yves Cottin, Catherine Quantin

**Affiliations:** a Department of General Medicine, UFR Sciences de Santé; b Biostatistics and Bioinformatics, University Hospital; c Laboratory of Cardiometabolic Physiopathology and Pharmacology, INSERM U866, UFR Sciences de Santé, Bourgogne Franche-Comté University, Dijon; d Economic Evaluation Unit, University Hospital, Montpellier, France; e Robert F. Wagner School of Public Service, New York University, New York, NY; f Department of Cardiology, University Hospital; g INSERM, CIC 1432; h Clinical Epidemiology/Clinical Trials Unit, University Hospital, Clinical Investigation Center, Dijon; i Biostatistics, Biomathematics, Pharmacoepidemiology and Infectious Diseases, INSERM, UVSQ, Institut Pasteur, Paris-Saclay University, Paris, France.

**Keywords:** elderly, myocardial infarction, primary care, rehospitalization, treatment

## Abstract

Rehospitalization after acute myocardial infarction (AMI) is common in elderly patients. It increases morbimortality and health care expenditures. The association between ambulatory care after discharge for AMI and rehospitalization has never been studied in France. We analyzed the impact of ambulatory care on rehospitalization of elderly patients (≥65 years) within 30 days after hospital discharge.

We conducted a nationwide population-based study of elderly patients hospitalized with a main diagnosis of AMI in France between 2011 and 2013. We excluded patients hospitalized for AMI in the previous year and those who died during the index hospitalization or within 30 days after discharge. The primary outcome was the first all-cause 30-day rehospitalization in an acute care hospital. Individual and neighborhood-level variables were compared among rehospitalized and nonrehospitalized patients. Determinants of 30-day rehospitalization were identified using logistic regression models.

Among the 624 eligible patients, 137 (22.0%) were rehospitalized within 30 days after discharge. In multivariate analyses, chronic kidney failure (odds ratio [OR] 1.88; 95% confidence interval [CI], 1.01–3.53) was an independent predictor of 30-day rehospitalization. We found no association among deprivation and spatial accessibility measures and 30-day rehospitalization. The purchase of lipid-lowering drugs prescription within 7 days after discharge was associated with a reduced risk of 30-day rehospitalization (OR 0.53; 95% CI, 0.36–0.79).

This study highlights the role of coordination among hospital and primary care physicians in post-AMI discharge and follow-up among elderly patients. Specifically, targeted interventions to reduce 30-day rehospitalizations should focus on patients with comorbidities and use of prescription drugs after hospital discharge.

## Abstract 2

Supplemental Digital Content is available in the text

## 1 Introduction

The transition from hospital to home is a high-risk period for elderly patients, commonly affected by multiple chronic comorbidities.^[1]^ Rehospitalizations within 30 days after hospital discharge, heretofore referred to as “30-day rehospitalizations,” are frequent adverse outcomes in this population^[2–4]^ with deleterious consequences including loss of autonomy, increased morbimortality^[5,6]^ and high socioeconomic costs.^[7,8]^ Thirty-day rehospitalization rates in elderly patients remain high whatever the initial hospitalization diagnosis: 14.7% in France and approximately 20% in the United States.^[4]^

Nearly one 30-day rehospitalization in 4 may be potentially avoidable.^[9]^ For this reason, reducing rehospitalizations has emerged as an important health policy goal among most OECD nations. Hospital-related risk factors have been well studied but the effects of ambulatory care after discharge are not well known.^[2,3,10,11]^ For some authors, improving postdischarge care may reduce rehospitalizations in elderly patients.^[12]^ Nevertheless, the results of transitional care programs are not consistent.^[13,14]^ Interventions to reduce rehospitalizations focus on patients’ early postdischarge follow-up with general practitioners (GPs)^[15–18]^ and home visits by nurses.^[19,20]^ Studies concentrate on isolated aspects of ambulatory care, whereas elderly patients frequently require multidisciplinary care with medical and paramedical primary care professionals. Little is known outside the context of clinical trials.^[18–20]^ To our knowledge, there is a lack of observational studies on the whole population of a country and the relationship between postdischarge ambulatory care and the risk of 30-day rehospitalization in elderly patients.

Acute myocardial infarction (AMI) is one of the leading initial diagnoses associated with higher rates of 30-day rehospitalization in elderly patients.^[4,21]^ Some authors have suggested that transitional care programs may be effective in reducing rehospitalizations after AMI.^[22]^ Moreover, American hospitals with discharge planning have lower rates of post-AMI 30-day rehospitalizations^[23]^ and American guidelines (ACCF/AHA STEMI) recommend a “posthospitalization plan of care” after AMI,^[24]^ The influence of postdischarge ambulatory care on 30-day rehospitalization after AMI has never been studied in France. Yet, such an investigation would lead to better understanding of the determinants of 30-day rehospitalization and suggest targeted strategies to reduce rehospitalizations. We hypothesize that improving postdischarge ambulatory care and identifying appropriate interventions after hospital discharge will reduce 30-day rehospitalization rates after AMI in France. Although some authors have shown the association between hospital and socioeconomic factors and 30-day rehospitalization after AMI,^[23,25]^ our aim here is to investigate the association between postdischarge ambulatory care and 30-day rehospitalization after discharge of elderly patients hospitalized for AMI, after adjusting for these factors.

## 2 Methods

### 2.1 Data sources

The SNIIRAM (Système National d’Information Inter-Régimes de l’Assurance Maladie) is the French national information system that contains individual, exhaustive, and linkable but anonymous data on healthcare use for approximately 77% of the French population.^[26]^ It aggregates data from

(1) The hospital discharge abstract database (Programme de Médicalisation des Systèmes d’Informations [PMSI]), which collects main and associated diagnoses (secondary events and comorbidities) encoded using the World Health Organization International Classification of Diseases and related health problems 10th revision (ICD-10), and procedures performed during hospital stays (in all public and private hospitals), using the common classification system for medical procedures (Classification commune des actes médicaux [CCAM]). The very good quality of this database has previously been evaluated and has enabled us to carry out epidemiological studies concerning hospitalized patients in France.^[4,27–32]^
(2) The reimbursement data for out-of-hospital care (consultations, procedures, drugs);
(3) The codes for long-term illnesses (LTIs), including coronary heart disease, that entitle patients to coverage without coinsurance under France's national health insurance program.^[33]^

Various control procedures are regularly conducted to ensure the quality of these data. The reliability of the SNIIRAM, including at first only the hospital database^[27–31,34]^ and recently the whole database,^[35,36]^ has been established in recent studies.

The EGB database (Échantillon Généraliste de Bénéficiaires) is an on-going representative, cross-sectional sample of the SNIIRAM, using a systematic 1/97th random sampling method.^[26]^ The data for this study were extracted from the EGB database. The French Institute of Health Data (IDS) approved this study (registration number 123, May 5, 2015).

### 2.2 Study design and setting

In France, the EGB database provides a representative sample of the French population and aggregates data on hospital discharge abstracts and reimbursement data for out-of-hospital care (consultations, procedures, drugs). We conducted a nationwide, population-based, retrospective study in France based on the French EGB database (including hospital and out-of-hospital care). All elderly patients hospitalized in France from January 2011 to December 2013 with a main diagnosis of AMI were identified in the EGB database using validated algorithms (ICD-10 codes I21, I22, I23).^[37]^ Among them, we included all patients admitted from home to acute inpatient hospitals (≥2 day stays). We considered rehospitalizations in short-term units only. We did not include rehospitalizations in long-term care facilities, such as aftercare and rehabilitation ward and long-term acute care. We excluded patients who died during the hospitalization or within the postdischarge study period (30 days for nonrehospitalized patients) because their healthcare use could not be determined. We also excluded patients hospitalized for AMI in the previous year to limit the potential effects of a previous hospitalization. The number of cases in the EGB database during the study period determined the sample size.

### 2.3 Outcomes

The first hospitalization for AMI between 2011 and 2013 was considered the index event. Discharge was the first discharge from an acute care hospital, whatever the patient's destination (home, aftercare and rehabilitation ward, transfer to another hospital). The primary outcome was the first all-cause 30-day rehospitalization in an acute care hospital, in the same or another hospital. That is, each patient was followed for 30 days after discharge. If any rehospitalization occurred during this period, the patient was considered to be rehospitalized.

### 2.4 Variables

For each patient, sociodemographic and medical data were collected at the index hospitalization: age, sex, place of residence, LTI status, length of stay, AMI type (segment elevation myocardial infarction [STEMI] *versus* non-ST-segment elevation myocardial infarction [NSTEMI]), stay in intensive care units, cardiac procedures performed (angioplasty with or without stent implantation, coronary artery bypass graft and other procedures on coronary arteries), and discharge destination. Main and associated diagnoses were used to analyze patients’ comorbidities and complications. Comorbid conditions were estimated with the Charlson Comorbidity Index (CCI), an independent predictor of mortality and recurrent AMI within 30 days after AMI.^[38]^ Congestive heart failure, acute and chronic kidney failure, diabetes, and atrial fibrillation were comorbidities of interest that we analyzed separately from the CCI. For rehospitalized patients, we examined the timing and the main diagnoses of rehospitalization.

Postdischarge ambulatory care included visits to GPs, cardiologists, and endocrinologists; home visits by nurses; cardiac rehabilitation; laboratory tests; further examinations; and prescription drug purchases (at least 1). We considered all prescription drugs purchased in private pharmacies after discharge, whether they were written by hospital or primary care physicians. We identified medications that conform to international practice guidelines following AMI,^[39]^ using the Anatomical Therapeutic Chemical classification system (ATC). We focused on beta-blockers (ATC code C07), antiplatelet/anticoagulant agents (B01), lipid-lowering drugs (C10), renin-angiotensin system blockers (C09), diuretics (C03), other cardiac therapies (C01), and antidiabetics (A10). We first reconstituted complete postdischarge ambulatory care during the observation period in each group: from the day of discharge to the day of rehospitalization for rehospitalized patients, from the day of discharge to 30 days later for nonrehospitalized patients. It is well known that nearly half of 30-day rehospitalizations after AMI occur within 7 days after discharge.^[40]^ Therefore, the observation period for rehospitalized patients is shorter than that for nonrehospitalized patients. We thus considered early postdischarge ambulatory care, within 7 days, for the 2 groups of rehospitalized and nonrehospitalized patients we compared.

The influence of socioeconomic factors and primary care accessibility on rehospitalization has been demonstrated.^[25,41,42]^ To adjust for these factors in our study, we added a neighborhood-level deprivation index to estimate patients’ socioeconomic status^[43]^ and a spatial accessibility indicator to account for the multiple dimensions of access to GPs, nurses, and pharmacists.^[44]^ Since these ecological indicators are not tied to individuals, they are collected at the local residence level (zip code).

### 2.5 Statistical analyses

We presented categorical variables as frequency distributions and continuous variables as means (with standard deviations). We then compared the characteristics of rehospitalized and nonrehospitalized patients using χ^2^ tests for categorical variables and Wilcoxon-Mann-Whitney tests for continuous variables. Next, we developed logistic regression models to analyze the impact of factors associated with 30-day rehospitalization, based on univariate analyses. In multivariate analyses, we introduced all individual variables considered significant in univariate analyses (*P* < .20) according to their clinical relevance based on correlation tests. We included age and CCI categorized into 3 groups according to the literature.^[18,38]^ We used backward selection.

The 30-day rehospitalization rate may vary according to the town of residence. To take this variability into account, we performed multilevel logistic regression models to test the association between individual and neighborhood-level variables and 30-day rehospitalization, taking into account the hierarchical structure of our data. We considered a 2-sided *P* < .05 as statistically significant. The results are presented as odds ratio (OR) and associated 95% confidence intervals. A forest plot showing the factors included in the multivariate regression analysis is also given. All analyses were performed using SAS, version 9.3 (SAS Institute, Cary, NC).

## 3 Results

**Figure 1:**
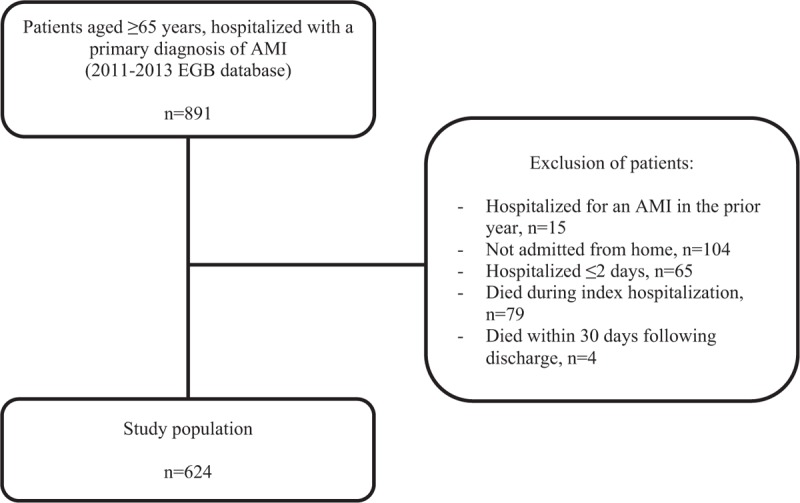
Study flow chart. AMI = acute myocardial infarction, EGB = Échantillon Généraliste de Bénéficiaires.

We identified a total of 891 patients aged ≥65 years, hospitalized in France with a main diagnosis of AMI in the 2011 to 2013 EGB database and included 624 in this study (Fig. 1). Among these, 137 (22.0%) were rehospitalized within 30 days after discharge. The mean time between discharge and rehospitalization was 11.9 (9.4) days. Rehospitalization diagnoses were cardiovascular for 56.2% with mostly chronic ischemic heart disease (19.7%) and heart failure (16.1%) (Supplementary appendix 1).

Patients’ baseline characteristics are shown in Table 1. The mean age was similar between rehospitalized and nonrehospitalized patients: 79.2 (7.7) and 78.1 (8.0) years, respectively (*P* = .15). Although the CCI and the AMI type did not differ significantly between the 2 groups, rehospitalized patients had a higher prevalence of congestive heart failure (*P* = .03), atrial fibrillation (*P* = .01), and chronic kidney failure (*P* = .02). A lower proportion of rehospitalized patients benefited from LTI status for coronary heart disease alone (*P* = .02), even though the proportion of patients with at least 1 LTI was the same in the 2 groups. No difference in socioeconomic status was observed. The length of stay and the procedures performed during the index hospitalization were similar (Table 2).

**Table 1:**
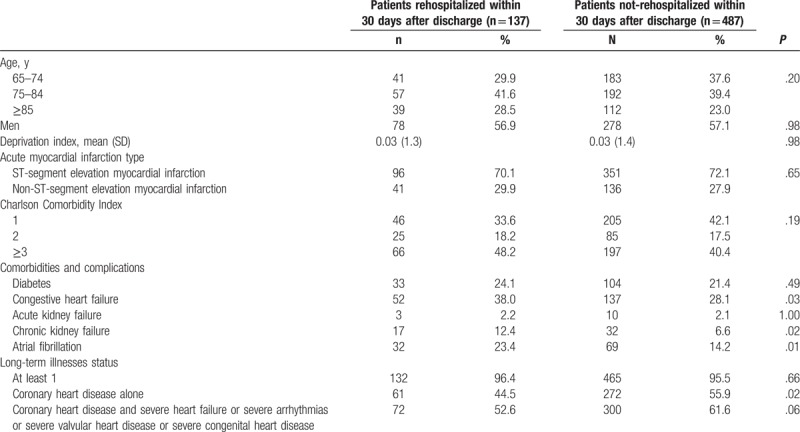
Patients’ baseline characteristics at the index hospitalization for acute myocardial infarction.

**Table 2:**
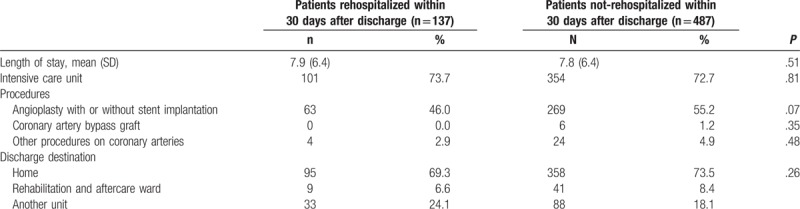
Index hospitalization management of elderly patients hospitalized for acute myocardial infarction.

Among the 137 thirty-day rehospitalized patients, 60 (43.8%) were rehospitalized within 7 days. Early postdischarge ambulatory care (within 7 days after discharge) did not differ significantly between rehospitalized and nonrehospitalized patients, except in their purchase of medications (Table 3). Rehospitalized patients purchased fewer antiplatelet/anticoagulant agents, lipid-lowering drugs, beta-blockers, and renin-angiotensin system blockers (*P* < .01).

**Table 3:**
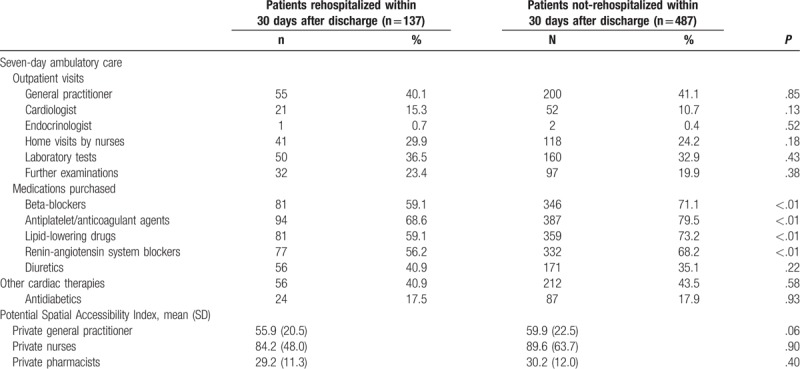
Early postdischarge ambulatory care after acute myocardial infarction in 30-day rehospitalized and nonrehospitalized elderly patients.

### 3.1 Factors associated with 30-day rehospitalization

**Figure 2:**
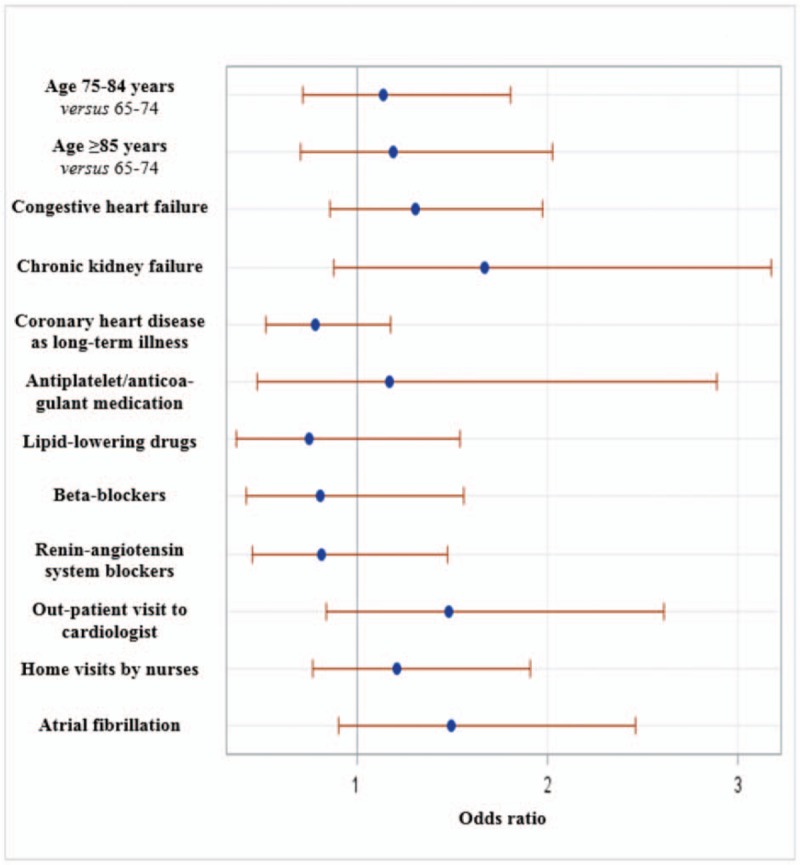
Forest plot of the factors included in the initial multivariate regression model.

**Figure 3:**
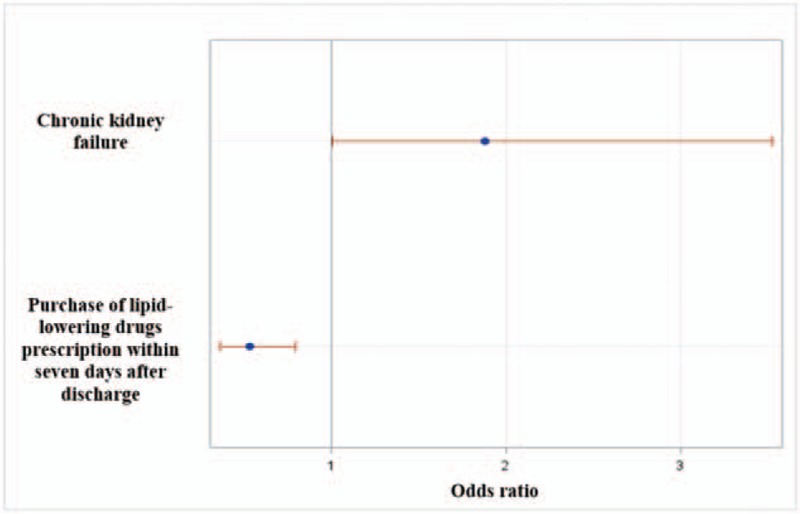
Significant results in multivariate analyses.

Factors included in the initial multivariate logistic regression model are shown in Figure 2. After backward selection, chronic kidney failure (OR = 1.88 [1.01–3.53]) was the only comorbidity (coded at the end of the index hospitalization) significantly associated with a higher risk of 30-day rehospitalization (Fig. 3). Early postdischarge medical follow-up showed no effect on 30-day rehospitalization. We found no association among deprivation and spatial accessibility measures and 30-day rehospitalization. The purchase of lipid-lowering drugs within 7 days after discharge was associated with a reduced risk of 30-day rehospitalization (OR = 0.53 [0.36–0.79]). These results were similar in multilevel logistic regression models.

## 4 Discussion

In this nationwide population-based study from a representative sample of the French population, we found that 22.0% of elderly patients (≥65 years) hospitalized for AMI were rehospitalized within 30 days after discharge. Regarding early postdischarge ambulatory care, we found that patients who purchased lipid-lowering drugs within 7 days after discharge were less likely to be rehospitalized within 30-days after discharge, but that the early ambulatory medical follow-up did not influence rehospitalization.

No study on rehospitalization after AMI has been published in France. Thus, we are unable to shed any comparative perspective on the evolution of rehospitalization rates for patients initially admitted for AMI. In the American national readmission database, the all-cause rehospitalization rate within 30 days after AMI was 14.7% in 2013.^[45]^ It decreased by 13% from 2009 to 2013.^[45]^ This rate is based on a nationally representative sample of the American population. In our study, we focused on elderly patients (65 years older) because they are at a higher risk of rehospitalization. Had we included Medicare patients only (≥65 years and younger severely disabled individuals), the 30-day rehospitalization rate after AMI would be higher (approximately 18% in 2013).^[46]^ Also, rehospitalization rates have decreased in the United States since the establishment of the Hospital Readmissions Reduction Program.^[45,46]^ This program has no equivalent in France which may explain, in part, the much higher rate of 30-day rehospitalization in France. In our study, we could not identify and exclude planned rehospitalizations, although they are common after AMI. This approach would have been interesting because unplanned rehospitalizations represent the majority of avoidable rehospitalizations. If we assume that 10% of the rehospitalizations were planned,^[7]^ 19.8% of our patients would have had unplanned rehospitalizations. This result is consistent with American studies^[23,38]^ and confirms the need for strategies to reduce unplanned 30-day rehospitalizations.

Few variables were consistently identified as predictors of post-AMI rehospitalization.^[47]^ This may reflect the complex management of these patients. In our study, chronic kidney failure was the only independent predictor of 30-day rehospitalization after AMI. The association between chronic kidney failure and 30-day rehospitalization has previously been described in NSTEMI patients^[48]^ and is now confirmed in elderly patients. Patients with chronic kidney failure may have more complications, cardiovascular or not, or poorer care which could lead to rehospitalization. They have to be identified early during the initial hospitalization as they require strengthened multidisciplinary care to limit rehospitalizations. We also found that almost half of 30-day rehospitalizations were not related to cardiovascular diagnoses. These results are consistent with other studies.^[40]^ This may suggest that rehospitalizations after AMI are heterogeneous in nature and may not be easily predicted, due to the high prevalence of comorbidities among these elderly patients. As rehospitalized patients benefited less from LTI status for coronary heart disease alone at the index hospitalization, we can also assume that they suffered from their first AMI.

We found that patients who purchased lipid-lowering drugs within 7 days after discharge were less likely to be rehospitalized within 30 days. International guidelines recommend their systematic use in post-AMI secondary prevention.^[39]^ We assume that the purchase of these medications may also be associated with better adherence to all treatments. Surprisingly, evidence-based post-AMI therapies were underused in our study, compared with others.^[49]^ A first hypothesis is that elderly patients may be undertreated due to comorbidities, multiple medications, or follow-up of lower quality. Another hypothesis is that there was poorer adherence to treatment, because we know that it decreases significantly with age and comorbidities.^[50]^ A third hypothesis is that we investigated medications over a short period of 7 days after discharge. Elderly patients could have continued taking the same medications they had before hospitalization and the failure to purchase may thus differ from the failure to follow treatment. Unfortunately, we were unable to distinguish prescribed medications from those purchased or to assess patients’ adherence. It would also have been interesting to distinguish antiplatelet and anticoagulant agents: the use of antiplatelet agents is recommended consistently after AMI, whereas anticoagulants are restricted to clinically indicated situations, such as atrial fibrillation. This distinction would require access to additional clinical data not available in our administrative database. Further research is needed to deepen our understanding of the role of treatments in 30-day rehospitalizations.

Postdischarge ambulatory care may be an opportunity to reduce 30-day rehospitalizations and improve patients’ outcomes. Several primary care professionals (GPs, nurses, pharmacists, …) may collaborate to promote greater adherence to treatment. In our study, early ambulatory medical postdischarge follow-up, however, showed no protective effect on 30-day rehospitalization. This is consistent with previous findings.^[51]^ Several reasons could account for this negative result. AMI is an acute disease for which short-term evolution is not the concern of primary care. As heart failure is, however, one of the most frequent reasons for 30-day rehospitalization after AMI, the potential long-term benefits of early GP follow-up should be studied more in detail in these elderly patients. In fact, Hernandez et al^[52]^ found that a GP follow-up within 7 days after discharge was associated with a reduced risk of 30-day rehospitalization in patients hospitalized for heart failure. Moreover, we found that nearly half of the patients were rehospitalized within 7 days, which is consistent with other studies.^[40,53]^ This early risk of rehospitalization emphasizes the importance of continuity of care. This post-discharge period must be anticipated with transitional care programs. The engagement of primary care professionals starting from the early postdischarge period may be important to prevent rehospitalizations.^[54]^

The strengths of this study include its original focus on postdischarge ambulatory care in everyday practice and the use of a representative sample of the French population, available from a nationwide exhaustive database combining hospital and ambulatory care data. The mean age and sex distribution of our sample were consistent with other studies in France^[49]^ and elsewhere.^[23]^ Regarding socioeconomic status, the mean deprivation index was similar to that of the general French population.^[43]^ Moreover, the validity of the EGB database for studying STEMI has been demonstrated^[55]^ and the same proportion of percutaneous coronary intervention was observed in the French main registry data (FAST-MI 2010).^[56]^

Some limitations must be acknowledged. The use of an administrative medical database limited the collection of socioeconomic data. Clinical information, as well, was restricted: information on patients’ left ventricular ejection fraction or on the extent of the coronary lesions would have enabled us to perform analyses adjusted for the AMI severity. Moreover, coding may sometimes be incomplete or incorrect and some comorbidities or procedures may have been underestimated. These problems are, however, unlikely because of the seriousness of the disease and the use of validated algorithms. Coding quality is checked by medical information professionals in each hospital to correct inaccurate diagnoses and to complete recorded comorbidities. Indeed, each French hospital's budget depends on the medical activity described in the PMSI database. Finally, some variables were too few in number and could not be considered for the analysis of post-discharge ambulatory care.

To conclude, our study suggests that a high proportion of elderly patients in France are rehospitalized within 30 days after discharge for AMI. The purchase of lipid-lowering drugs prescription within 7 days after discharge was associated with a reduced risk of 30-day rehospitalization. We can postulate that the poor discharge planning and insufficient care coordination between hospital and community-based professionals in France hamper strategies to reduce rehospitalizations. Targeted strategies to reduce 30-day rehospitalizations in elderly patients should focus on patients’ comorbidities and treatments. Further research is needed regarding the importance of early primary care follow-up for patients hospitalized for AMI.

## Acknowledgments

The authors thank the project committee members from the Directorate for healthcare provision at the Ministry of Health (Adeline Townsend, Adrien Dozol et Agnès Solomiac [bureau R5 « Évaluation, méthodes et modèles », Direction Générale de l’Offre de Soins]) and the national agency for the management of hospitalization data (Véronique Sauvadet, Florence Pinelli, Nathalie Rigollot, Laëtitia Chossegros, Eric Ekong, Philippe Demey et Catherine Le Gouhir [Agence Technique de l’Information sur l’Hospitalisation]). The authors also thank Suzanne Rankin for reviewing the English.

## Author contributions

**Conceptualization:** Claire Zabawa, Marianne Zeller, Grégoire Mercier, Victor Rodwin, Yves Cottin, Catherine Quantin.

**Formal analysis:** Jonathan Cottenet.

**Methodology:** Jonathan Cottenet, Marianne Zeller, Grégoire Mercier, Victor Rodwin, Yves Cottin, Catherine Quantin.

**Supervision:** Catherine Quantin.

**Validation:** Claire Zabawa, Catherine Quantin.

**Writing – original draft:** Claire Zabawa, Victor Rodwin, Catherine Quantin.

**Writing – review and editing:** Claire Zabawa, Jonathan Cottenet, Marianne Zeller, Grégoire Mercier, Victor Rodwin, Yves Cottin, Catherine Quantin.

## Supplementary Material

Supplemental Digital Content
