# Supplementary material for: Thirty-day rehospitalizations among elderly patients with acute myocardial infarction: Impact of postdischarge ambulatory care
Source: Medicine (Baltimore). 2018 Jun 15;97(24):e11085. doi: 10.1097/MD.0000000000011085 (PMC6023939; doi:10.1097/MD.0000000000011085)
Supplement: Supplemental Digital Content [file medi-97-e11085-s001.docx]

**Appendix**

**Supplementary appendix 1. The 10 most common primary diagnoses for 30-day rehospitalization following discharge for acute myocardial infarction (n=137)**

| Primary diagnosis of rehospitalization | n | % |
| --- | --- | --- |
| Chronic ischemic heart disease | 27 | 19.7 |
| Heart failure | 22 | 16.1 |
| ST elevation and non-ST elevation myocardial infarction | 11 | 8.0 |
| Angina pectoris | 10 | 7.3 |
| Pain in throat and chest | 6 | 4.4 |
| Encounter for other aftercare and medical care | 4 | 2.9 |
| Atrial fibrillation and flutter | 3 | 2.2 |
| Pneumonia, unspecified organism | 3 | 2.2 |
| Encounter for other post procedural aftercare | 3 | 2.2 |
| Encounter for care involving renal dialysis | 3 | 2.2 |
